# Supplementary material for: Involvement of the AKT Pathway in Resistance to Erlotinib and Cabozantinib in Triple-Negative Breast Cancer Cell Lines
Source: Biomedicines. 2023 Aug 28;11(9):2406. doi: 10.3390/biomedicines11092406 (PMC10525382; doi:10.3390/biomedicines11092406)
Supplement: Supplementary file 1 [file biomedicines-11-02406-s001.zip › biomedicines-2503732-supplementary gata.pdf]

Supplemental Information For:

Involvement of the AKT pathway in resistance to erlotinib and cabozantinib in triple negative breast cancer cell lines.

Cory Lefebvre, Sierra Pellizzari, Vasudeva Bhat, Kristina Jurcic, David W. Litchfield, Alison L. Allan

1. Supplementary Materials and Methods

1.1. Flow cytometry analysis

To assess whether the development of resistance was associated with an increase in the breast cancer stem cell (ALDH^{high}/CD44^{+}/CD24^{-}) population, aldehyde dehydrogenase (ALDH) activity and expression of CD24 and CD44 by resistant cell models were assessed with flow cytometry. Using the ALDEFLUOR™ Kit (StemCell Technologies, Vancouver, BC, Canada), cells were incubated with either DEAB (diethylaminobenzaldehyde) or ALDEFLUOR™ reagent prior to incubation with 20µL of phycoerythrin (PE)-conjugated anti-human CD44 antibody (clone 510, BD Biosciences, Mississauga, ON, Canada) and 5µL of PE-CyTM7 conjugated anti-human CD24 antibody (clone ML5, BD Biosciences). Cells were washed with PBS and resuspended in 500µL of ALDEFLUOR™ assay buffer. All samples were stored on ice in the dark and analyzed on an FC500 flow cytometer (Beckman Coulter, Miami, FL, USA). For gating, forward and side scatter were used to eliminate cell debris and doublets from the analysis. At least 10,000 events were collected for each replicate. Cells were first gated for ALDH activity using the DEAB negative control, and then ALDH^{high} cells were gated for CD24 and CD44 expression using appropriate controls.

The MDR (Multidrug Resistance) flow cytometry kit (Abcam, Cambridge, UK) was used to assess whether ATP-binding cassette (ABC) efflux transporter/drug pump activity contributes to the development of resistance in our cell models. Cells were incubated with one of the provided inhibitors (1.5µmol ABCG2-inhibitor Novobiocin; 300nmol ABCB1-inhibitor Verapamil; 750nmol ABCC1-inhibitor MK-571) prior to incubation with fluorescent efflux green detection reagent. For analysis, at least 10,000 events were collected for each replicate. Using forward and side scatter, cells were gated to eliminate cell debris and cell doublets. Viable cells were gated for the propidium iodide-negative (PI-) population using appropriate controls (unlabeled and PI only labeled cells). Median fluorescent intensity (MFI) from inhibited and uninhibited conditions to measure fluorescent shift as result of inhibition of ABC pump activity. All data were analyzed using FlowJo (v10.8.0).

1.2. Detailed LC-MS/MS Data Acquisition and Processing Methodology

1.2.1. Preparation of Phosphoproteomic and Proteomic Samples

SILAC-labeled TNBC cells were treated with inhibitors or DMSO vehicle control for 24 h prior to collection of protein lysates for phosphoproteomic and proteomic sample preparation. This included 2 biological replicates with "Light"-control and "Heavy"-erlotinib conditions; and 2 biological replicates with labels swapped. To collect protein lysates, cells in 100mm plates were washed twice with ice-cold PBS (Wisent) and scraped into 0.1mL of GdmCl lysis buffer (6M Guanidium Chloride, 100mM Tris pH 8.5, 10 mM tris(2-carboxyethyl)phosphine (TCEP), 40 mM 2-chloroacetamide (CAA)). Lysates were heated at 95°C for 5 min, cooled on ice for 15 min, then sonicated in a water bath (4 cycles of 30s on:30s off) and heated at 95°C for 5 min. Lysates were centrifuged for 30 min at 3,500xg and transferred to clean tubes for storage at -80°C with an aliquot used to measure protein concentration using NanoDropTM One Spectrophotometer (Thermo). Protein lysates from heavy labeled cells were precipitated using 4 sample volumes of methanol in 1 sample volume of chloroform and 3 sample volumes of MS-grade H_2O (Millipore-Sigma) over ice. Samples were centrifuged at 14,000xg centrifugation for 2 min at 4°C to pellet

precipitated protein, followed by removal of the aqueous layer using vacuum aspiration. Ice-cold methanol was added to wash the pellet, followed by centrifugation, removal of the aqueous layer and air-drying to near-dryness. Protein pellets were reconstituted in tri-fluoro ethanol (TFE) digestion buffer (50mM ammonium bicarbonate, 5% (v/v) (TFE) to a concentration between 0.5 to 1.0 mg/mL with 1:100 Trypsin/Protease LysC (Promega V5073) and were digested for 4 h at 37°C with agitation in a shaking incubator. An additional aliquot of 1:100 trypsin (V5111; tri-fluoro ethanol) was added to samples before incubation overnight for 18 h at 37°C with agitation. Digested peptides were desalted using C18 StageTip (Empore, Saint Paul, MN, USA), then concentrated in a vacuum centrifuge until near-dryness and reconstituted in 0.1% (v/v) formic acid (FA) for proteomic samples.

Aliquots of digested peptides were reserved for phosphopeptide enrichment and were adjusted to 3.2M KCl, 150mM KH_2PO_4, 50% (v/v) ACN and 6% (v/v) trifluoroacetic acid (TFA). Bulk 10 μM titanium dioxide (TiO_2) beads (5020-75010, GL Sciences, Tokyo, Japan) was prepared in loading buffer (80% ACN (v/v), 6% TFA (v/v)) at a ratio of 10:1 (w/w) (1mg of TiO_2 beads per 100 μg of peptides) in micro-centrifuge tubes and incubated for 5 min at 40°C with end-over-end rotation. Samples were then spun at 3,500 x g for 1 min, after which the supernatant was removed. TiO_2 beads were then washed four times by adding 1mL of wash buffer (60% ACN (v/v), 1% TFA (v/v)) and mixing vigorously using a vortex genie, followed by centrifugation at 3,500x g for 1 min. TiO_2 beads were then reconstituted in 500μL of transfer buffer (80% ACN (v/v), 0.5% FA (v/v)), incubated at room temperature for 30 s, and then transferred onto a single-layer C18 StageTip (Empore). StageTips were centrifuged at 500xg for 5 min at RT. Phosphopeptides were eluted from StageTips with 100uL elution buffer (40% ACN (v/v), 15% NH_4OH (25%, HPLC grade)), centrifuged at 500xg for 3 min at 4°C, and elutes were collected in clean micro-centrifuge tubes. Samples were concentrated in a vacuum centrifuge until about 20uL remained and acidified with 10% TFA. Samples were then loaded unto double-layer SDB-RPS StageTips (Empore), which were pre-equilibrated with ACN then 30% methanol + 0.2% TFA followed by 0.2% TFA. SDB-RPS StageTips were washed twice 0.2% TFA followed by centrifugation at 500 xg for 5 min at RT. Phosphopeptides were eluted from StageTips with SDB-RPS elution buffer (80% ACN, 5% NH4OH (25%, HPLC grade) and collected into clean micro-centrifuge tubes following centrifugation at 500 x g for 5 minutes at room temperature. Collected samples were concentrated in a vacuum centrifuge until near-dryness and reconstituted in 0.1% (v/v) formic acid.

1.2.2. Preparation of Kinomics Samples

SILAC-labeled TNBC cells were treated with inhibitors or DMSO vehicle control for 24 h prior to collection of cell pellets for kinomics sample preparation. Two biological replicates comprised "Light"-labeled cells treated with control and "Heavy"-labelled cells treated with erlotinib, with one biological replicate label-swapped such that "Light"-labelled were treated with erlotinib and "Heavy"-labelled were treated with control. Cell plates were washed twice with ice-cold PBS over ice and scraped into PBS. Samples were centrifuged at 2,000xg for 10 min; the supernatant was aspirated, and the cell pellet was flash-frozen in dry ice. Cell samples were processed by the Lunenfeld Tanenbaum Research Institute (Toronto, Canada) as described below.

Cell pellets were lysed in 4:1 (v/w) volume of lysis buffer [50 mM HEPES pH 7.5, 150 mM NaCl, 0.5% Triton X-100, 1 mM EDTA, 1 mM EGTA, 10 mM NaF, 2.5 mM Na_3VO_4, 1x Protease Inhibitor Cocktail (Sigma-Aldrich cat#P8340), 1x Phosphatase Inhibitor Cocktail 2 (Sigma-Aldrich cat#P5726), Phosphatase Inhibitor Cocktail 3 (Sigma-Aldrich cat#P0044)]. Cells were further mechanically lysed by passing 12 times through an 18G needle attached to a syringe. Lysates were cleared in a centrifuge at 16,000 x g at 4°C for 20 min. Protein concentration determined using the Bio-Rad Bradford Protein Assay kit (Bio-Rad cat#5000006). Prior to application on beads, paired cells samples with light and medium labels were combined at a ratio of 1:1 for each SILAC experiment.

The broad-spectrum bisanilino pyrimidine kinase inhibitor CTx-0294885 (ref PMID:23692254) (MedKoo Biosciences, Inc.) was covalently coupled to NHS-activated

Sepharose 4 Fast Flow beads (GE Healthcare, cat# 17-0906-01) to generate iCTx beads. The 50% iCTx bead slurry was washed 3 times with 1 ml lysis buffer. After each wash, beads were centrifuged at 500 x g and the supernatant was removed. After all washes, beads were resuspended in lysis buffer at 50% bead slurry.

Combined lysates were transferred into tubes containing iCTX beads and incubated in an end-over-end shaker for 30 minutes at 4°C. After incubation with beads, each sample was spun down at 500 x g and the supernatant was discarded. The beads were next washed once with lysis buffer and 3 times with 50 mM ammonium bicarbonate buffer (ABC). (After each wash, beads were spun down at 500 x g and supernatant discarded). One µg of trypsin was added to each sample in 50mM ABC, and the samples were incubated on a rotator at 37°C overnight. After centrifugation at 500 x g for 1 minute the supernatant was transferred to a new tube. Beads were rinsed with 50 mM ABC prior to the supernatant combined with that of the overnight digestion. Trypsin (0.5 µg) was added to the combined supernatants and the sample was incubated on a rotator at 37°C for 4 hours. After 4 hours, 50% formic acid was added for a final concentration of 4% formic acid. Any remaining beads in the sample were spun down at 16,000 x g for 5 minutes. The cleared supernatant was transferred to a new tube and the peptides were lyophilized in a vacuum evaporator and stored at -80°C.

1.2.3. Assessment of SILAC label incorporation

To evaluate SILAC label incorporation efficiency, a sample from TNBC cells cultured with medium labels (Arg-6, Lys-4) was lysed and processed as a proteome sample described above and LC-MS/MS parameters for data acquisition followed those described below, with data acquisition performed on an Orbitrap Elite mass spectrometer. Raw spectra were analyzed using MaxQuant v1.6.2.10, Max Planck Institute of Biochemistry, Munich) [26,27] and searched against the Swissprot- Uniprot sequence database (homo sapiens). Default settings [27] were used with minor changes: multiplicity of 2 was selected with heavy labels designated as Lys4 and Arg6, carbamidomethyl (C) was set as a fixed modification while oxidation (M), acetylation (protein N-terminal), deamidation (NQ) and phosphorylation (STY) were set as variable modifications. Proteins and peptides were identified using a target-decoy approach with a reversed database and with a 1% FDR for peptide spectrum matches and protein matches. The heavy and light intensity values from MS1 quantitation were used to calculate H/L ratios. Incorporation inefficiency for labeled amino acids was evaluated by calculating the proportion of quantified peptides that had an H/L ratio greater than two.

1.2.4. LC-MS/MS Data Acquisition

Proteomic and phosphoproteomic peptide samples were loaded onto an ACQUITY UPLC Symmetry C18 NanoAcquity, 10K, 2G V/M, 180 µm x 20 mm, 100A, 5 µm trapping column (Waters Corporation, Milford, MA) via a Waters NanoAcquity UPLC, at a flow rate of 10 µL/min for 4 minutes using 99% buffer A (0.1% formic acid) and 1% buffer B (acetonitrile, 0.1% formic acid). After trapping, peptides were eluted onto the analytical column for separation. Flow was established at 300 nL/min for the ACQUITY UPLC Peptide BEH C18 NanoAcquity Column 10K psi, 130Am 1.7 µm x 25 mm, which was held at 35˚C. Peptides from proteome samples were separated using a 290-minute non-linear gradient. The gradient initial condition was 5% buffer B. Buffer B was then increased to 7.5% over 1 minute, 25% over 179 minutes, 32.5% over 40 minutes, 60% over 20 minutes, 98% over 5 minutes, then held at 98% for 5 minutes, reduced to 2% over 5 minutes, then held at 2% for 5 minutes, increased to 98% over 5 minutes, then held at 98% for 5 minutes, reduced to 5% over 5 minutes and finally held at 5% for 15 minutes for re-equilibration to the initial condition. This gradient builds in a saw-tooth washing stage to clean the column. Peptides from phosphoproteome samples were separated using a 130-minute non-linear gradient. The gradient initial condition was 5% buffer B. Buffer B then increased to 7.5% over 1 minute, 25% over 59 minutes, 32.5% over 14 minutes, 60% over 6 minutes, 98% over 5 minutes, then held at 98% for 5 minutes, reduced to 2% over 5 minutes, then held

at 2% for 5 minutes, increased to 98% over 5 minutes, then held at 98% for 5 minutes, then reduced to 5% over 5 minutes, and finally held at 5% for 15 minutes for re-equilibration to the initial condition.

The LC system was directly connected to a NanoFlex (Thermo Electron Corp., Waltham, MA) nanospray ionization source with a source voltage of 2.4 KV and was interfaced to an Orbitrap Elite VelosPro Ion Trap - Orbitrap mass spectrometer. The mass spectrometer was controlled by Xcalibur software (Thermo, v2.7.0) and operated in the data-dependent mode using an FT/IT/CID Top 20 scheme. The MS scan recorded the mass-to-charge ratios (m/z) of ions over the range of 400–1450 in FT (resolution of 120,000 at m/z 400), positive ion, profile, full MS mode using a lock mass (445.120025 m/z). The 20 most abundant multiply charged ions were automatically selected for subsequent collisional induced dissociation in ion trap mode (IT/CID) with an isolation width of 2.00 Da, rapid scan rate, centroid mode, with charge state filtering allowing only ions of +2, +3 and +4 charged states. Normalized collision energy was 35 and precursor ions were then excluded from further CID for 30 seconds.

Kinomic peptide samples were loaded onto a nano-HPLC (High-performance liquid chromatography) system coupled to an Orbitrap Fusion™ Lumos™ Tribid™ mass spectrometer for data-dependent acquisition (DDA) LC-MS/MS. Nano-spray emitters were generated from fused silica capillary tubing, with 75µm internal diameter, 365µm outer diameter and 5-8µm tip opening, using a laser puller (Sutter Instrument Co., model P-2000, with parameters set as heat: 280, FIL = 0, VEL = 18, DEL = 2000). Nano-spray emitters were packed with C18 reversed-phase material (Reprosil-Pur 120 C18-AQ, 1.9 µm) resuspended in methanol using a pressure injection cell. The sample in 5% formic acid was directly loaded at 400 nl/min for 20 minutes onto a 75 µm x 15 cm nano-spray emitter. Peptides were eluted from the column with an acetonitrile gradient generated by an Eksigent ekspert™ nanoLC 425, and analyzed on an Orbitrap Fusion™Lumos™Tribrid™. The gradient was delivered at 200 nl/min from 2.5% acetonitrile with 0.1% formic acid to 30% acetonitrile with 0.1% formic acid using a linear gradient of 120 minutes. This was followed by a 8 min gradient from 30% acetonitrile with 0.1% formic acid to 80% acetonitrile with 0.1% formic acid. After, there was a 5 min wash with 80% acetonitrile with 0.1% formic acid, and equilibration for another 23min at 2.5% acetonitrile with 0.1% formic acid. The total DDA protocol is 150 minutes. The MS1 scan had an accumulation time of 50 ms within a mass range of 400–1500Da, using orbitrap resolution of 120000, 60% RF lens, AGC target of 125% and 2400 volts. This was followed by MS/MS scans with a total cycle time of 3 seconds. Accumulation time of 50 ms and 33% HCD collision energy was used for each MS/MS scan. Each candidate ion was required to have a charge state from 2-7 and an AGC target of 400%, isolated using orbitrap resolution of 15,000. Previously analyzed candidate ions were dynamically excluded for 9 seconds.

1.2.5. LC-MS/MS Data Processing

Mass spectrometric data were analyzed using MaxQuant (v1.6.2.10, Max Planck Institute of Biochemistry, Munich) [61,62] and searched against the Human Swissprot-Uniprot FASTA database (homo sapiens), which contained 42,146 entries. Default settings [62] were used with minor changes: multiplicity of 2 was selected with heavy labels designated as Lys4 and Arg6, carbamidomethyl (C) was set as a fixed modification while oxidation (M), acetylation (protein N-terminal), deamidation (NQ) and phosphorylation (STY) were set as variable modifications. For quantification, unmodified peptides as well as those with fixed and variable modifications, excluding phosphorylation (STY), were included. "Match Between Runs", using default matching parameters for respective pairs of samples between biological replicates, was selected. Proteins and peptides were identified using a target-decoy approach with a reversed database and with a 1% FDR for peptide spectrum matches and protein matches.

MaxQuant analysis outputs were interpreted using Perseus (v1.6.15.0, Max Planck Institute of Biochemistry, Munich) [63]. For the analysis of proteome data, the "proteinGroups.txt" file was first processed to remove entries marked as reverse decoy

database hits, contaminants, and protein identifications that were only matched to modified peptides. MaxQuant-generated normalized ratios were used, and the log base 2 of the normalized ratios were calculated for each protein. For analysis of the phosphoproteome data, the “Phospho (STY)Sites.txt” file was first processed to remove entries marked as reverse decoy database hits and contaminants and filtered to only contain phosphorylation sites with localization probability greater than 0.75. Phosphorylation sites were expanded in Perseus to identify quantified sites being derived from single-, double- and triple-phosphorylated peptides. Rows where no quantification was present were removed. The analysis of the kinome data was conducted in the same way as the proteome data described above. To identify the kinases, UniProt IDs were matched to the UniProtKS/Swiss-Prot list of human protein kinases. (https://www.uniprot.org/docs/pkinfam).

2. Supplementary Figures and Tables

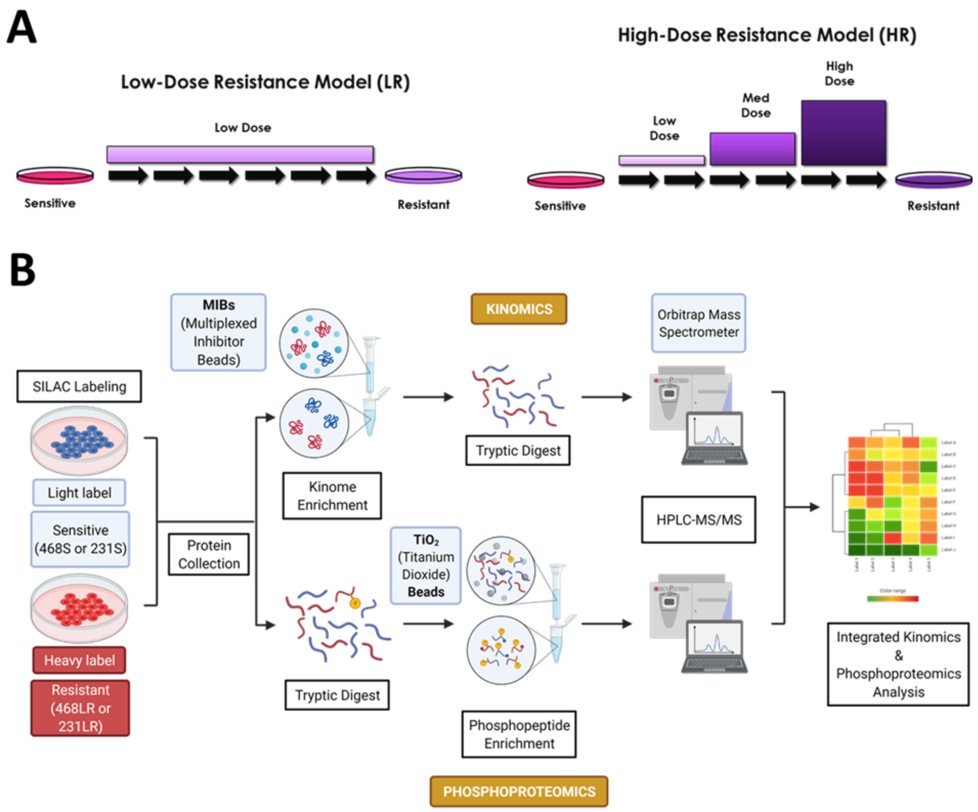

Supplemental Figure S1. Schematics of resistance development and LC-MS/MS workflow. (A) Schematic representation of the development of low-resistance (LR) and high-resistance (HR) cell line models through long-time exposure of parental MDA-MB-468 of MDA-MB-231 TNBC cells to erlotinib or cabozantinib, respectively. (B) Integrated phosphoproteomic and kinomics workflow to investigate development of erlotinib- and cabozantinib-resistance. For relative quantification between resistant (low-resistance model) and sensitive (control) conditions, MDA-MB-468S, MDA-MB-468LR, MDA-MB-231S and MDA-MB-231LR cells were labeled using SILAC (stable isotope labeling by amino acids in cell culture) metabolic labeling. Samples were subjected to HPLC-MS/MS to generate proteomic, phosphoproteomic and kinomics datasets which were analyzed assessing for changes in protein expression, phosphorylation, kinase expression and activity changes.

Supplemental Table S1. Antibodies used for immunoblotting.

Antigen	Phosphosites	Clone [1]	Dilution [2]	Source
Anti-Human ABCB1 (MDR1)	--	Mono (E1Y7S)	1/1000	Cell Signaling Technology Cat#13978S
Anti-Human ABCC1 (MRP1)	--	Mono (D7O8N)	1/1000	Cell Signaling Technology Cat#14685S
Anti-Human ABCG2 (B	--	Poly	1/1000	Cell Signaling Technology Cat#4477S
Anti-Human AKT1	--	Mono (9Q7)	1/1000	Thermo Fisher Scientific Cat# AHO1112
Anti-Human Phospho-AKT1	Ser473	Mono (14-6)	1/1000	Thermo Fisher Scientific Cat# 44-621G
Anti-Human Phospho-AKT1	Thr308	Poly	1/1000	Thermo Fisher Scientific Cat# 44-602G
Anti-Human c-Met	--	Mono (22H22L13)	1/1000	Thermo Fisher Scientific Cat# 700261
Anti-Human Phospho-c-Met	Tyr1234, Tyr 1235	Mono (3D7)	1/1000	Cell Signaling Technology Cat#3129
Anti-Human E-Cadherin	--	Mono (36)	1/1000	BD Biosciences Cat#610181
Anti-Human EGFR	--	Mono (1F4)	1/1000	Cell Signaling Technology Cat# 2239, RRID: AB_331373
Anti-Human Phospho-EGFR	Tyr1086	Mono (1240C)	1/1000	R&D Systems Cat# MAB8967
Anti-Human Ep-CAM	--	Mono (E1-44)	1/1000	Abcam Cat#ab32392

Anti-Human ERK1/2	--	Mono (K.913.4)	1/1000	Thermo Fisher Scientific Cat# MA5-15134, RRID: AB_10982335
Anti-Human Phospho-ERK1/2	Thr202, Tyr204	Mono (S.812.9)	1/1000	Thermo Fisher Scientific Cat# MA5-15173, RRID: AB_11009630
Anti-Human N-Cadherin	--	Mono (EPR1791-4)	1/1000	Abcam Cat#AB76011
Anti-Human Vimentin	--	Mono (V9)	1/1000	Sigma-Aldrich Cat#MAB3400
Anti-Human β-actin	--	Mono (SP124)	1/2000	Sigma-Aldrich Cat# SAB5500001

[1] Clonality of the antibody (Mono–monoclonal, Poly–polyclonal) with specified clone in parentheses for monoclonal antibodies. [2] Primary antibodies diluted at specified ratio in 5% BSA (Bovine Serum Albumin) in 1X TBS-T (Tris-buffered saline + 0.1% Tween-20).

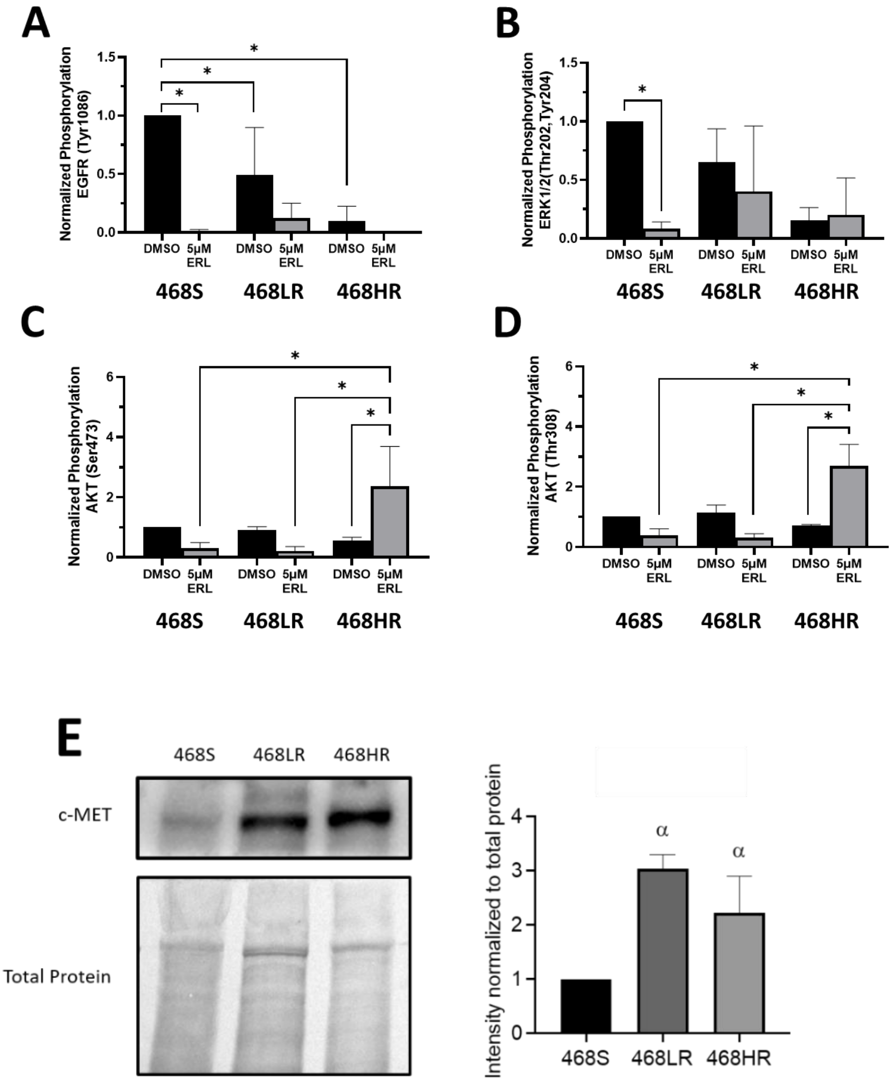

Supplemental Figure S2. Triple negative human MDA-MB-468 breast cancer cells demonstrate changes in EGFR signaling in response to treatment depending on sensitivity vs. resistance to erlotinib. Densitometric quantification of immunoblots (n = 3) from *Figure 1D*. Ratios of phosphorylated to total protein expression of *(A)* EGFR, *(B)* ERK1/2, *(C)* AKT1-Ser473, and *(D)* AKT1-Thr308 in response to 5µM erlotinib (ERL) or DMSO, normalized to total protein loading control (Amido Black Stain) and to respective 468S DMSO control. * = significantly different than respective DMSO control. *(E)* Protein expression of c-MET in 468S, 468LR and 468HR breast cancer cells lines. Representative immunoblots are shown on the left, with densitometric quantification (n=3) shown on the right. Band intensity was normalized to total protein (amido black staining). α = significantly different than 468S.

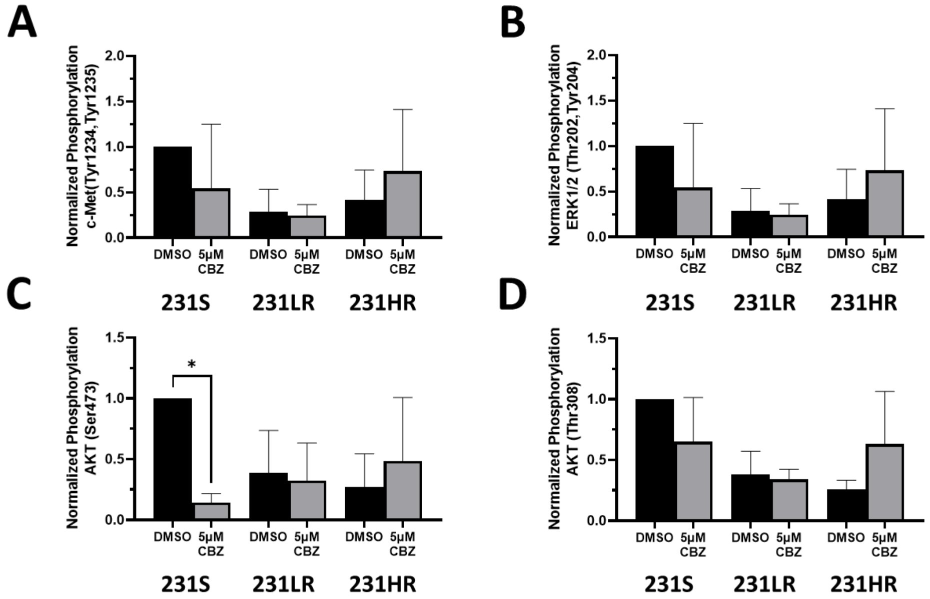

Supplemental Figure S3. Triple negative human MDA-MB-231 breast cancer cells demonstrate changes in AKT signaling in response to treatment depending on sensitivity vs. resistance to cabozantinib. Densitometric quantification of immunoblots (n=3) from Figure 2D. Ratios of phosphorylated to total protein expression of (A) c-MET, (B) ERK1/2, (C) AKT1-Ser473, and (D) AKT1-Thr308 in response to 5µM cabozantinib (CBZ) or DMSO, normalized to total protein loading control (Amido Black Stain) and to respective 231S DMSO control. * = significantly different than respective DMSO control.

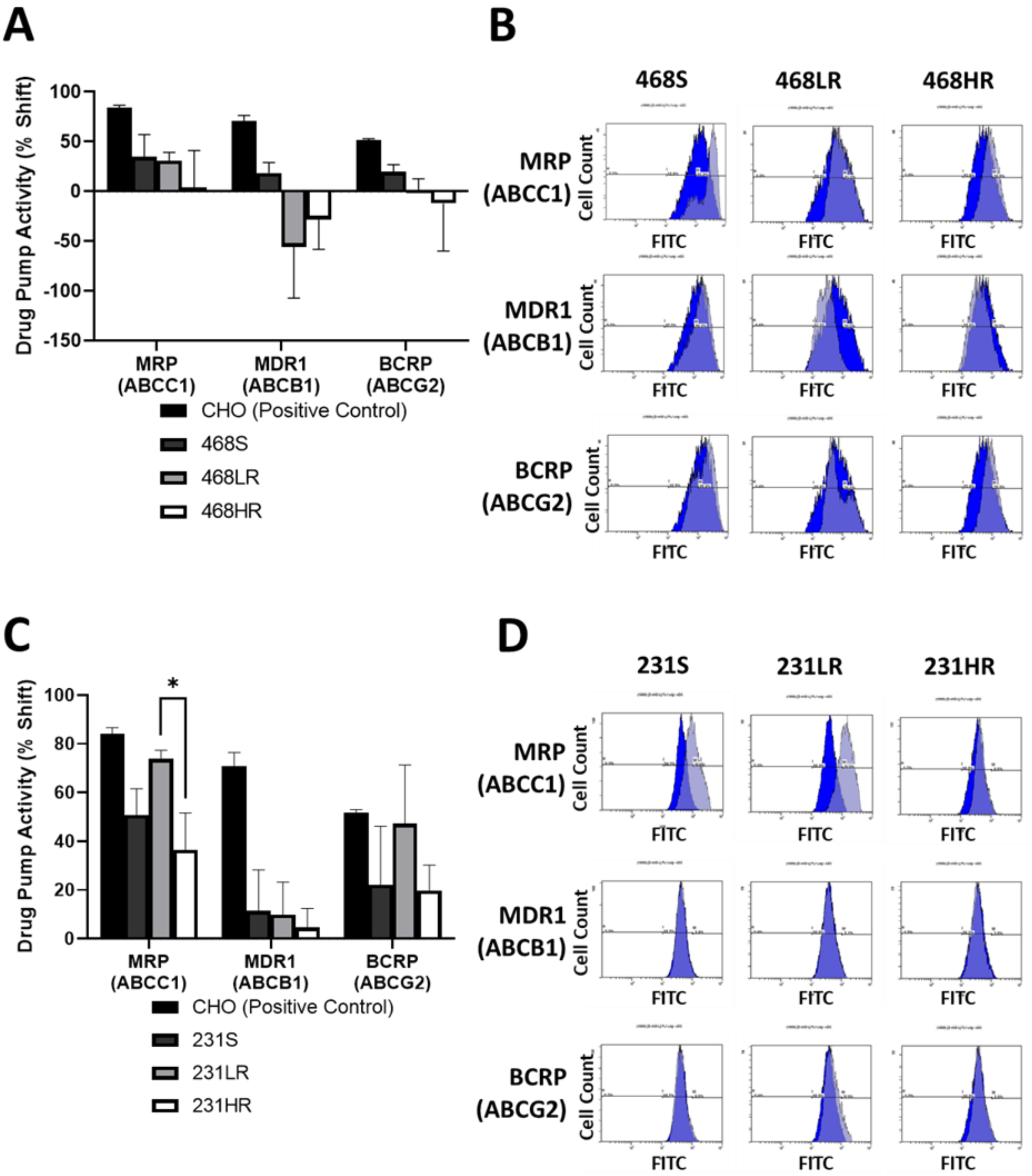

Supplemental Figure S4. Triple negative breast cancer cells do not have altered drug efflux pump activity in the presence of acquired resistance to erlotinib or cabozantinib. rug pump activity for ABCC1, ABCB1 and ABCG2 was measured by flow cytometry using the Abcam MDR kit. Drug pump activity in (A,B) erlotinib-resistant (468LR, 468HR) and erlotinib-sensitive (468S), (C,D) cabozantinib-resistant (231LR, 231HR) and cabozantinib-sensitive (231S) cells, with CHO cells used as a positive control. (A,C) Quantitative data of % shift in fluorescence intensity in response to treatment with specific drug pump inhibitors (n = 3). (B,D) Representative fluorescent histograms of inhibitor-treated (pale blue) overlayed with untreated (darker blue) control for each cell model.

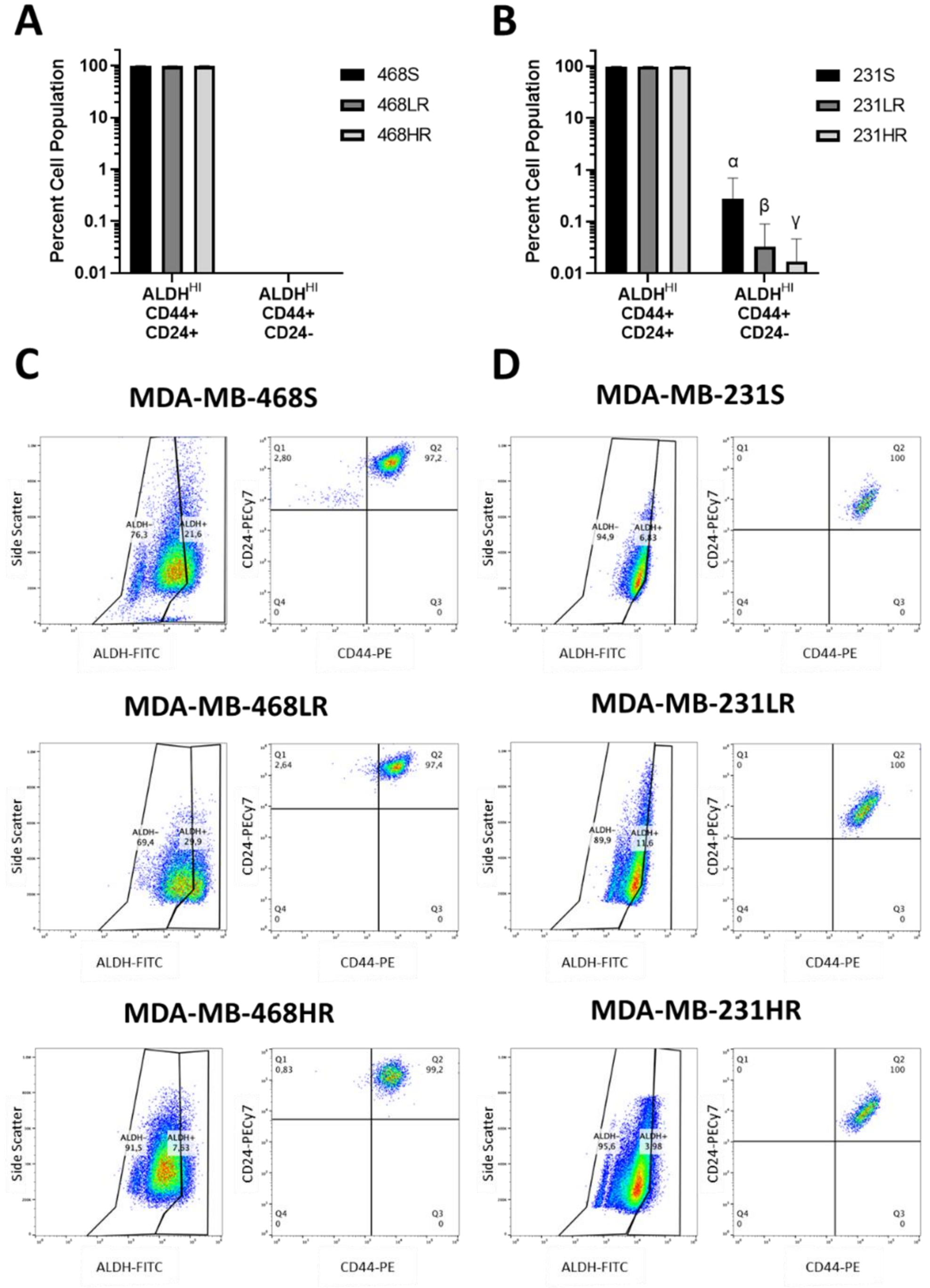

Supplemental Figure S5. Triple negative breast cancer cells do not demonstrate a change in cancer stem cell (CSC) population in the presence of acquired resistance to erlotinib or cabozantinib. Aldehyde dehydrogenase (ALDH) activity and CD44/CD24 cell surface expression were assessed by flow cytome-try, with the breast CSC phenotype defined as ALDHhigh/CD44+/CD24-. (A,B) Quantitative assessment of the proportion of CSC cell populations with high ALDH activity or CD44+/CD24- phenotype (A) erlo-tinib-resistant (continued) (468LR, 468HR) or erlotinib-sensitive (468S) cells; and (B) cabozantinib-resistant (231LR, 231HR) or cabozantinib-sensitive (231S) cells (n = 3). Define statistical notations on graphs. (C,D) Representative flow cytometry histograms of ALDH activity (left panels) and CD44/CD24 expression (right panels) for each cell model.

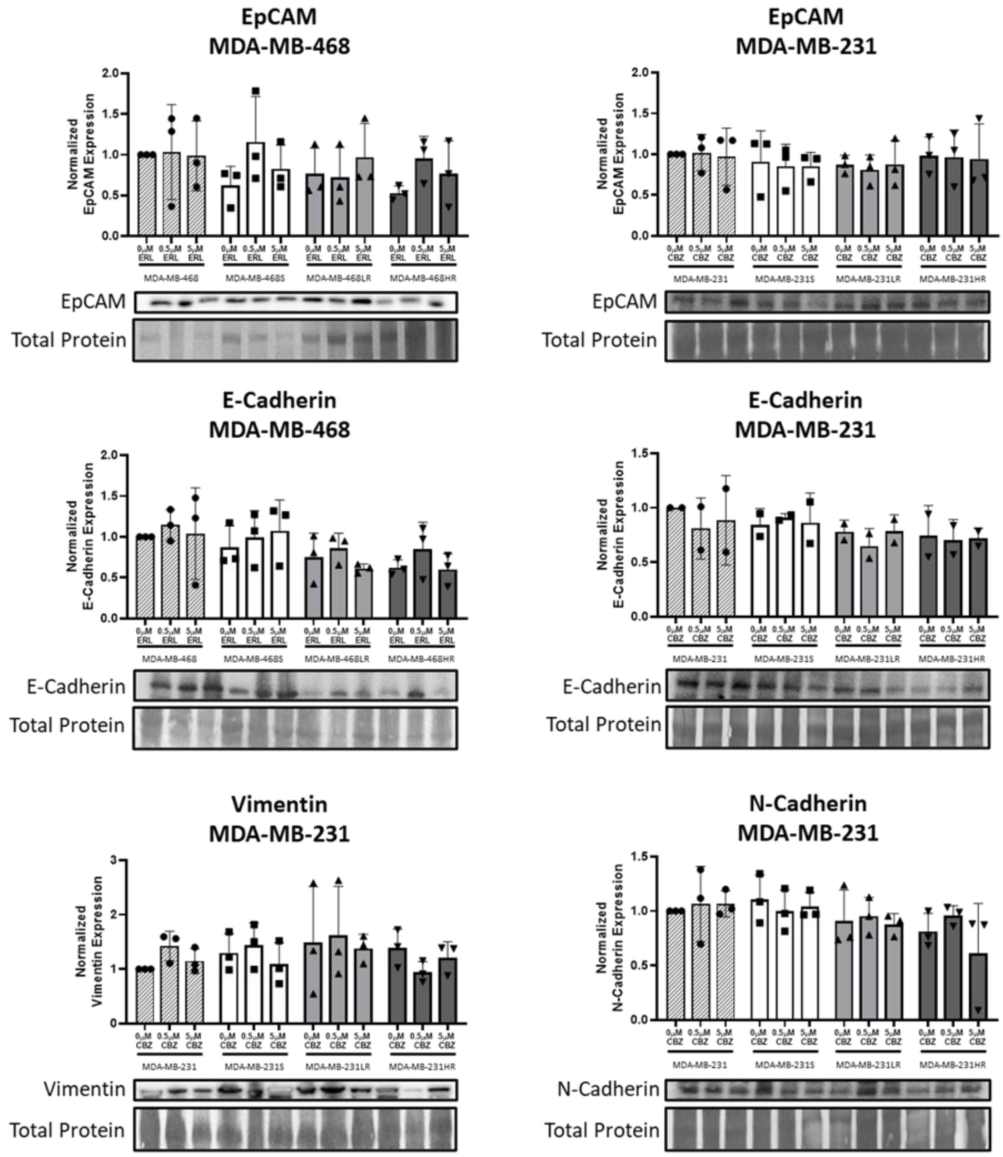

Supplemental Figure S6. Triple negative breast cancer cells do not demonstrate a change in epithelial-to-mesenchymal (EMT) markers in the presence of acquired resistance to erlotinib or cabozantinib. Expression of (A-D) epithelial markers EpCAM and E-Cadherin or (E-H) mesenchymal markers vimentin and N-cadherin was assessed via immunoblotting of cell lysates from resistant (468LR, 468HR, 231LR, 231HR), sensitive (468S, 231S) or parental (468, 231) cell models treated with erlotinib (ERL) or cabozantinib (CBZ). Quantified expression (n = 3/condition) was normalized to total protein loading control and to respective parental cells treated with DMSO.

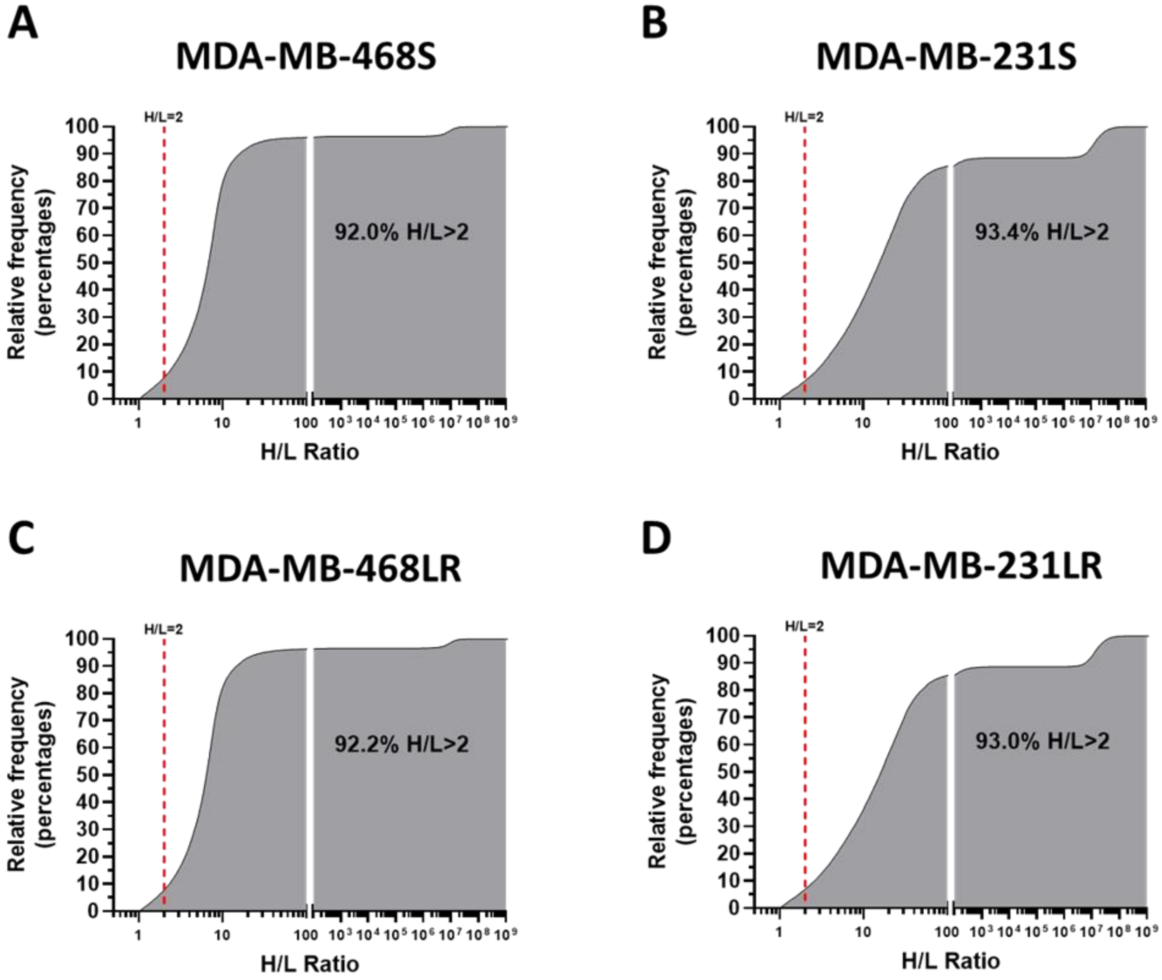

Supplemental Figure S7. Frequency distribution of SILAC label incorporated peptides in medium-labeled sensitive and resistant MDA-MB-231 and MDA-MB-468 cells. To verify SILAC label incorporation, aliquots of (A,B) sensitive 468S and 231S and (C,D) low-resistant 468LR and 231LR cells cultured with SILAC-Medium Label (Arginine-6, Lysine-4) were analyzed for label incorporation as described in the Materials and Methods. Distribution is representative of all quantified peptides with intensity of medium label (H) to non-label (L). The red dashed line indicates an H/L ratio of 2.

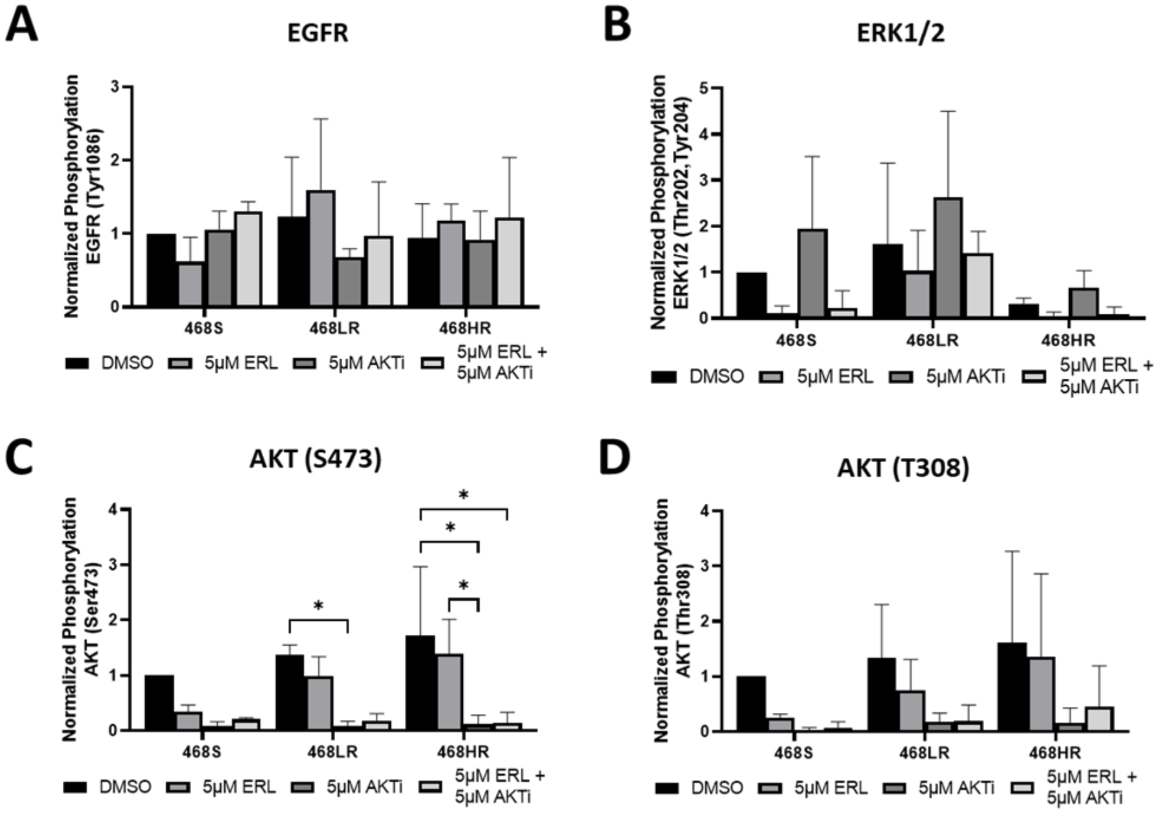

Supplemental Figure S8. Resistant and sensitive triple negative human MDA-MB-468 breast cancer cells demonstrate changes in AKT1 signaling in response to addition of AKT inhibitor VIII with erlotinib. Densitometric quantification of immunoblots (n = 3) from Figure 8B. Ratios of phosphorylated to total protein expression of (a) EGFR, (b) ERK1/2, (C) AKT1-Ser473, and (D) AKT1-Thr308 in response to 5μM erlotinib (ERL), 5μM AKT Inhibitor VIII (AKTi), 5μM erlotinib + 5μM AKT Inhibitor VIII, or DMSO, normalized to total protein loading control (Amido Black Stain) and to respective 468S DMSO control. * = significantly different than respective DMSO control.

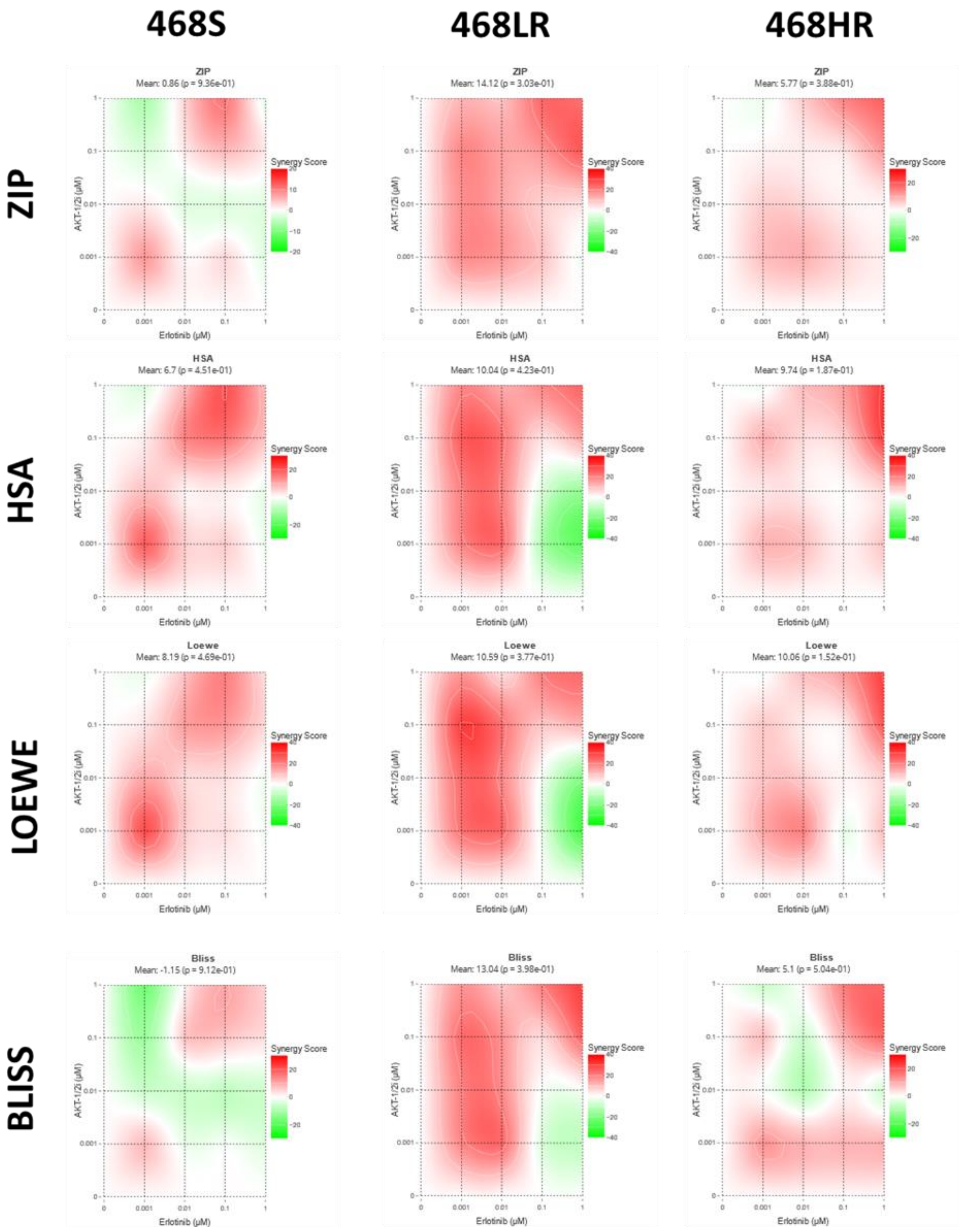

Supplemental Figure S9. Synergy scores of erlotinib and AKT Inhibitor VIII in MDA-MB-468 TNBC cells. Contour maps of the ZIP, HSA, Loewe and Bliss synergy scores for the matrix of erlotinib and AKT-1/2i combinations for erlotinib-resistant (468LR, 468HR) and erlotinib-sensitive (468S) cells. Graphs were generated using Synergy Finder web portal [66].

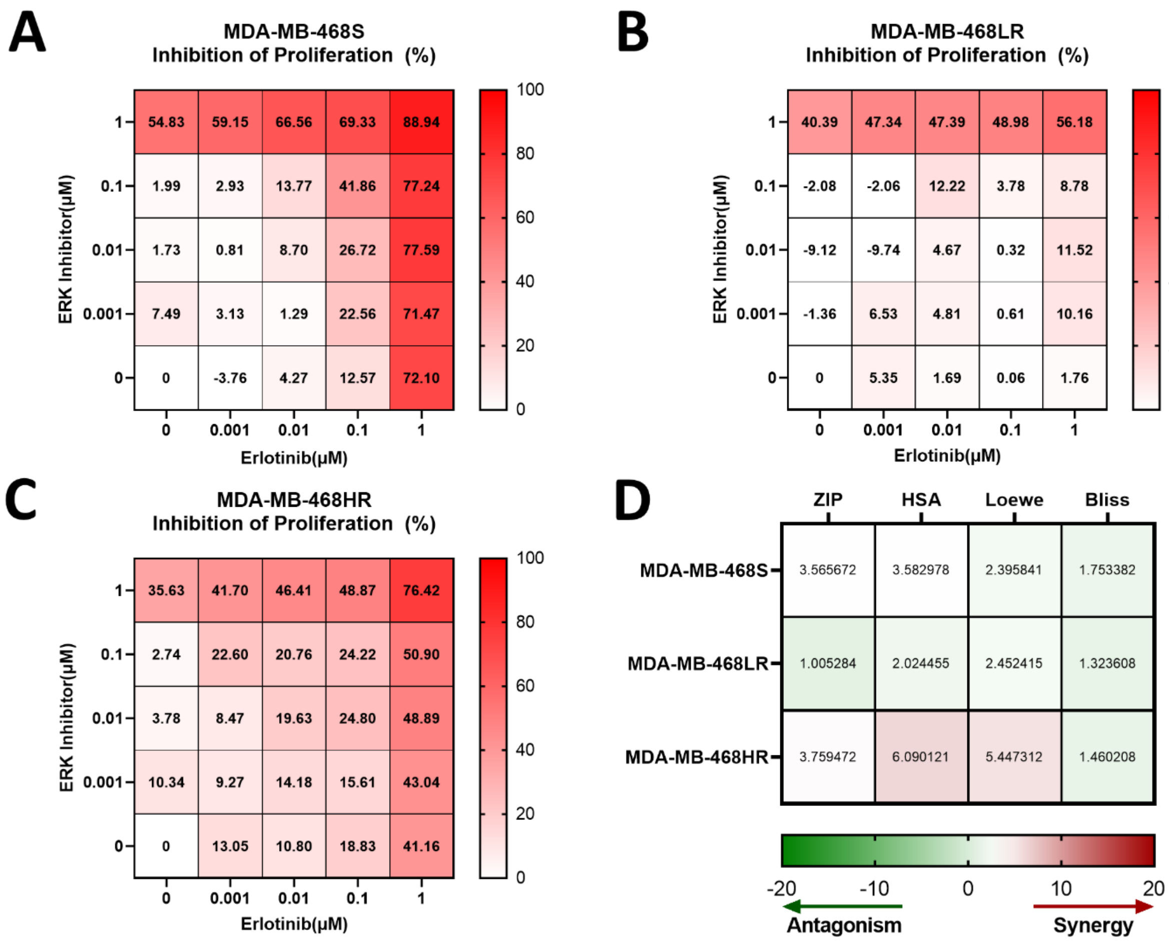

Figure S10. Combination targeting of ERK with erlotinib is weakly synergistic in erlotinib resistant MDA-MB-468 cells. (A-C) Heatmaps of percent inhibition for the erlotinib + ERK inhibitor temuterkib combination treatments of the sensitive (A: 468S) and resistant (B: 468LR and C: 468HR) cell lines. Inhibition was determined by measuring the surviving fraction of colony forming assays with matrix of concentrations (0µM, 0.001µM, 0.01µM, 0.1µM, and 1µM) for each inhibitor in each inhibitor combination. (D) Heatmap of the synergy scores (ZIP, HSA, Loewe and Bliss) of the inhibitor combinations erlotinib + ERK inhibitor temuterkib treatment for the resistant and sensitive cell lines.
